# Well-Being and Performance of Nursery Pigs Subjected to Different Commercial Vaccines Against Porcine Circovirus Type 2, *Mycoplasma hyopneumoniae* and *Lawsonia intracellularis*

**DOI:** 10.3390/vaccines12111242

**Published:** 2024-10-31

**Authors:** Caio Abércio Silva, Marco Aurélio Callegari, Cleandro Pazinato Dias, Kelly Lais de Souza, Gabrieli Souza Romano, Luciana Fiorin Hernig, Ricardo Tesche Lippke, Rutger Jansen, Fernando Lopes Leite, Fernando Filipe, Rafael Humberto de Carvalho

**Affiliations:** 1 Department of Animal Science, Center of Agrarian Sciences, State University of Londrina, Londrina 86057-970, Brazil; rafael.carvalho@uel.br; 2 Akei Animal Research, Fartura 18870-970, Brazil; contato@akei.agr.br (M.A.C.); cleandro@cleandrodias.com.br (C.P.D.); tecnico@akei.agr.br (K.L.d.S.); gabrieli.romano@akei.agr.br (G.S.R.); 3 Boehringer Ingelheim do Brasil, Sao Paulo 04795-100, Brazil; luciana.hernig@boehringer-ingelheim.com (L.F.H.); ricardo.lippke@boehringer-ingelheim.com (R.T.L.); filipe.fernando@boehringer-ingelheim.com (F.F.); 4 Boehringer Ingelheim Vetmedica GmbH, Bingerstrasse 173, 55216 Ingelheim am Rhein, Germany; rutger.jansen@boehringer-ingelheim.com; 5 Boehringer Ingelheim Animal Health, 3239 Satellite Blvd, Duluth, GA 30096, USA; fernando.leite@boehringer-ingelheim.com

**Keywords:** biomarkers of inflammation, ileitis, piglet welfare, post-vaccination behavior

## Abstract

**Background/Objectives:** Vaccination is a strategy in pig farming for the control of several pathogens, but commercial vaccines may have detrimental side effects. This study aimed to evaluate the effects of commercial vaccines on the control of porcine circovirus type 2 (PCV2), *Mycoplasma hyopneumoniae* (Mhp), and *Lawsonia intracellularis* (*L*. *intracellularis*) and their potential side effects on welfare, behavior, acute inflammation biomarkers (C-reactive protein and haptoglobin), and the performance of piglets during the nursery phase. **Methods:** A total of 240 piglets, both female and castrated males, with an average weight of 6.3 ± 0.9 kg were subjected to four treatments: T1-FLEXcombo^®^ (Ingelvac^®^CircoFLEX and Ingelvac^®^MycoFLEX) + Enterisol^®^ Ileitis; T2-FLEXCombo^®^ + Porcilis^®^ Ileitis; T3-Porcilis^®^ PCV M HYO + Porcilis^®^ Ileitis; and T4-FLEXCombo^®^ + 0.9% saline solution. This study measured therapeutic interventions, body condition score, behavioral changes, rectal temperature, and inflammation biomarkers post-vaccination. **Results:** The T3 group required more therapeutic interventions and exhibited a 23.1% higher incidence of thin body condition (*p* < 0.05) and 10 times more animals with depressed behavior than T1 (*p* < 0.05). The piglets vaccinated for *L. intracellularis* (T2 and T3) had rectal temperatures exceeding 39.7 °C post-vaccination, significantly higher than in T1 (*p* < 0.05). The T1 animals showed five times more positive behavior traits 24 h after vaccination (*p* < 0.05). Touch response was 29% lower in the T2 and T3 groups, and the lying down behavior was higher in these groups compared to T1. Additionally, 41.7% of the T3 animals exhibited a sitting posture 48 h after vaccination. Higher serum C-reactive protein and haptoglobin levels were observed in T3 (*p* < 0.05) at 24 and 48 h post-vaccination. Feed intake was higher in T1 compared to T3 between 29 and 35 days of age. It is important to note that this study did not measure immune responses to the pathogens and did not include challenge tests, and therefore, it does not assess which vaccine is superior in pathogen control. **Conclusions:** The vaccine programs resulted in similar zootechnical performance. However, T1, T2, and T4 showed better effects on piglet welfare and behavior compared to T3.

## 1. Introduction

The performance, health, and welfare of commercially raised pigs are related to the zootechnical, environmental, and sanitary management practices [1]. Due to the demands of society and the laws related to ethics in animal production, the pig industry has directed efforts to meet these needs, which are subject to adjustments [2,3,4].

In commercial pig farming, challenges related to animal health and welfare are present in all phases of production. However, weaning remains one of the most critical moments for animal welfare [5]. Currently, due to legal and production efficiency issues, weaning is increasingly occurring at ages older than 21 days [6].

At weaning, piglets are exposed to stress conditions, such as transfer to a new environment, contact with other piglets, and disruption of milk feeding, which is replaced by a completely different diet in terms of its physical and nutritional presentation. These factors, associated with the low immune status and the incomplete digestive enzymatic maturity that the animals have [7,8,9,10], make piglets more susceptible to the development of systemic, respiratory, nervous, and/or intestinal pathological conditions that, in the short- or medium-term, promote growth retardation and increase mortality [11]. Associated with weaning, vaccination of piglets has become a widespread practice aimed at minimizing the possible risks caused by agents such as porcine circovirus type 2 (PCV2), *Mycoplasma hyopneumoniae* (Mhp), and *Lawsonia intracelullaris* (*L. intracellularis*) [12,13,14].

Mhp is the causative agent of Enzootic Pneumonia and one of the main primary agents involved in respiratory diseases of pigs. This pathogen can affect piglets in the nursery and growing and finishing phases [15]. PCV2 has been associated with several clinical diseases that are collectively termed porcine circovirus disease (PCVD), including subclinical infection (PCV-2-SI), systemic diseases (PCV-2-SD), reproductive (PCV-2-RD), and porcine dermatitis and nephropathy syndrome (PDNS) [16,17]. *L. intracellularis*, in turn, is responsible for ileitis or proliferative enteropathy, a disease ubiquitously present in animals in the growth and finishing phase. This can occur in acute forms (PHE), most often causing diarrhea with the presence of subclinical or chronic blood, causing zootechnical losses and an increased mortality rate [18].

Although the benefit of vaccination against these agents is evident, with positive results already in the nursery phase, there are commercial vaccines that are often associated with the induction of undesired effects [19], such as the occurrence of local or systemic reactions. These immediately affect the welfare of the piglets, causing typical conditions of prostration, hyperthermia, and reduced feed intake [20]. Thus, they affect the adaptation of these animals in the nursery phase, with consequences on performance, which may extend until the ages close to slaughter [21].

Vaccine reactions are closely associated with the nature of the adjuvants used, as these can trigger local or systemic responses depending on how they activate the immune system [22]. This exacerbated activation can lead to depression, pyrexia, and impaired well-being [20]. Additionally, injectable parenteral administration is also a reason for discomfort and pain, which is why other routes of administration, such as oral administration, have gained prominence to minimize this discomfort [23].

These conditions can be verified by measuring serum levels of acute phase proteins with the biomarkers haptoglobin and C-reactive protein (CRP) [24]. These support, in addition to the determination of the induced inflammatory status, the general level of pig welfare [25]. The concentrations of these proteins may remain altered for approximately 14 days [26] and can be measured through analyzing blood, saliva, and/or hair [27,28].

From this perspective, animal welfare indicators, based on the measurement of physiological and behavioral parameters, have also been commonly used in pig farming to estimate the quality of life of piglets in the face of challenges [29]. In this regard, the behavioral and postural evaluation of pigs after vaccination has been used to determine the state of comfort and well-being [30,31]. A positive post-vaccination welfare indication involves piglet interest in human interaction and feed intake, which can be easily verified, thus demonstrating the natural behavior of pigs [31]. The combination of lack of activity and anorexia after vaccination indicates a reduction in welfare and may result in a compromised zootechnical performance.

The objective of this study, therefore, was to measure the effects of commercial vaccines frequently used in the control of the pathogens PCV2, Mhp, and *L. intracellularis* and their consequences on the welfare and performance of piglets during the nursery phase.

## 2. Materials and Methods

The procedures adopted in this study were performed according to the practices approved by the Ethics Committee on the Use of Research Animals of Akei Animal Research (protocol number 022/20) and by the international animal welfare guidelines. A detailed table listing the parameters measured, their respective methods, and indications is provided as Supplementary Material (Table S1).

### 2.1. Animals

A total of 240 PIC piglets (Camborough x AG 337^®^) were used, females and castrated males, weaned at 22 days of age, with a mean initial weight of 6.351 ± 0.871 kg, from a commercial farm positive for PCV2, Mhp, and *L. intracellularis*.

The piglets were housed in 40 pens (six animals of the same sex per pen), which had an area of 2.55 m^2^, a fully slatted floor, a nipple pendulum drinker (with adjustable height), and a linear feeder with six feeding spaces. All animals received water and feed ad libitum throughout the experimental period (42 days). The feeding of the animals was based on the use of a program with four diets formulated to meet the minimum nutritional requirements established by Rostagno et al. [32].

### 2.2. Experimental Design

The piglets were randomly distributed in blocks according to sex and weaning weight into four treatments, with ten replicates and six animals per pen totaling 60 piglets per treatment, considering that, for the performance parameters, the pen was the experimental unit and, for the other parameters, the piglet represented the replication. The experimental treatments (Table 1) corresponded to the use of different commercial vaccines aimed at the prevention of PCV2, Mhp, and *L. intracellularis*.

The piglets were discriminated as T1, T2, T3, and T4. T1, T2, and T4 received the vaccines FLEXCombo^®^: (Ingelvac^®^CircoFLEX and Ingelvac^®^MycoFLEX) (Boehringer Ingelheim Vetmedica Inc., St. Joseph, MO, USA) for immunization against PCV2 and Mhp. The T3 treatment animals were vaccinated with the Porcilis^®^ PCV M HYO vaccine (MSD Animal Health, Boxmeer, the Netherlands). All groups were vaccinated, according to the manufacturers’ recommendations, with a single dose of 2 mL administered intramuscularly with disposable 27 × 0.7 mm sterile needles on the right side of the neck on the day of weaning (corresponding to 22 days of age).

For the prevention of ileitis caused by *L. intracellularis* and to evaluate the sole impact of this vaccination, these vaccines were administered at 29 days of age, and the animals belonging to treatment T1 received the vaccine Enterisol^®^ Ileitis (Boehringer Ingelheim Vetmedica Inc., St. Joseph, MO, USA), administered orally by drench following the manufacturer’s recommendation (Table 1). The piglets belonging to treatments T2 and T3 received the vaccine Porcilis^®^ Ileitis (MSD Animal Health, Boxmeer, the Netherlands), and T4 received 0.9% saline solution. All animals in the treatments that received the vaccines via the parenteral route (i.e., except for T1) were immunized with 2 mL of the vaccines via the deep intramuscular route (injectable with disposable 27 × 0.7 mm sterile needles) on the left side of the neck.

### 2.3. Data Collection

The individual weighing of the piglets and the measurement of feed intake, after deducting leftovers and losses, were performed at weekly intervals. The data obtained allowed for the calculation of daily feed intake, daily weight gain, and feed conversion, which were expressed according to each nutritional phase throughout the entire experimental period. To assess animal health and welfare, the piglets were observed daily and individually by a single observer. Any animal considered unhealthy was examined by a veterinarian, removed, and housed in hospital pens for specific treatment. One animal from T1 and one from T4 were removed due to encephalitis and treated separately. Additionally, one animal from T4 died from sudden death, and another animal from T3 was removed due to regular absence of feed intake/anorexia. For the performance variables, the removal of these animals impacted the average by adjusting for feed leftovers and weighing the remaining animals in the pen to calculate the pen average. For the other variables analyzed, the removal of these animals did not have a significant impact, and they were treated as missing data in the analysis.

A diarrhea score [33] was measured daily throughout the experimental period, according to a classification of feces: 0—stools of normal consistency, 1—pasty, 2—moderately fluid, 3—aqueous.

From day 22 to 36 of age, the depression and flank scores of the piglets were measured daily and individually. The evaluation of the depression score was adapted according to the methodology described by Rossi et al. [34], which is classified as follows: 0—lively, alert, and responsive animals; 1—animals that were standing and isolated but quickly showed response to stimulus; 2—animals that were standing and isolated with a bowed head, may present muscle weakness and respond with a delay to the stimulus; and 3—depressed animals, lying down and reluctant to get up.

The flank score was determined according to Spiehs, Shurson, and Johnston [35], where a score of 1 indicated an animal with a normal abdomen and full and round flanks, while a score of 2 considered an animal with a little full intestine and flat flanks, and a score of 3 indicated severely lean animals with empty flanks.

Additionally, 20 piglets per treatment, two per pen, half males and half females, were randomly chosen for measures of rectal temperature 1 h before and 8 and 24 h after vaccination against the agent *L. intracellularis*. At all times, the same piglets were evaluated.

To evaluate the welfare of the animals subjected to the vaccine against PCV2, Mhp, and *L. intracellularis*, the behavior and posture of the piglets were analyzed based on the protocol described by Weimer et al. [30], in which all piglets were classified as touched, oriented, or not oriented. Animals classified as not oriented were further divided into four postures, standing, sitting, supported, and lying down, and into two behaviors, eating and drinking. This procedure was performed 24 and 48 h after vaccination. For the *L. intracellularis* vaccine, behavioral observations were performed before vaccination and 4, 8, 24, 48, and 72 h after vaccination and were always measured by the same observer.

The serum levels of haptoglobin and C-reactive protein were evaluated by the Pig CRP ELISA Kit (ab205089, Abcam, VIC, Australia) and Pig Haptoglobin ELISA kit (ab205091, Abcam, VIC, Australia), respectively. For this determination, 20 piglets (half males and half females) were randomly selected and subjected to blood collection by puncture (5.0 mL syringe, 40 × 10 mm needle) from the vessels of the neck region in vacutainer tubes without anticoagulant at three time points: a few minutes before vaccination against *L. intracellularis* (D7) and 24 h (D8) and 48 h (D9) after vaccination. The samples were kept at 4 °C overnight before serum recovery by centrifugation, which was stored at −20 °C until analysis.

### 2.4. Statistical Analysis

The Box and Whisker package was used to identify potential outliers; however, no animals were classified as outliers in this study. The normality of the distribution of the data was analyzed using the Kolmogorov–Smirnov and Lilliefors test and the Shapiro–Wilk W test (*p* > 0.05). The homogeneity of variances was verified using Levene’s test. Data following a normal parametric distribution underwent analysis of variance using the General Linear Model, with the model considering block, sex, and treatment effects. The means from this analysis were further evaluated using Tukey’s test. Non-normally distributed quantitative or categorical data were compared using the Kruskal–Wallis test followed by Dunn’s post-test. Both analyses were performed using Statistic for Windows^®^ software, version 10.0 (StatSoft, Tulsa, OK, USA. 2011). For the tests, a *p*-value equal to or less than 0.05 was considered significant, and a *p*-value between 0.05 and 0.10 was considered a trend.

## 3. Results

### 3.1. Zootechnical Performance and Clinical Signs

There was no significant difference (*p* ≥ 0.05) in mean body weight, daily weight gain, or feed conversion between treatments in any of the nursery phases or in the cumulative period (Table 2). However, during the pre-initial phase II (29 to 35 days of age), the animals vaccinated with the T1 immunization protocol had a significant (*p* < 0.05) increase in daily feed intake compared to the animals in treatment T3. This difference in feed intake was not observed in the other periods evaluated (*p* ≥ 0.05).

**Figure 1:**
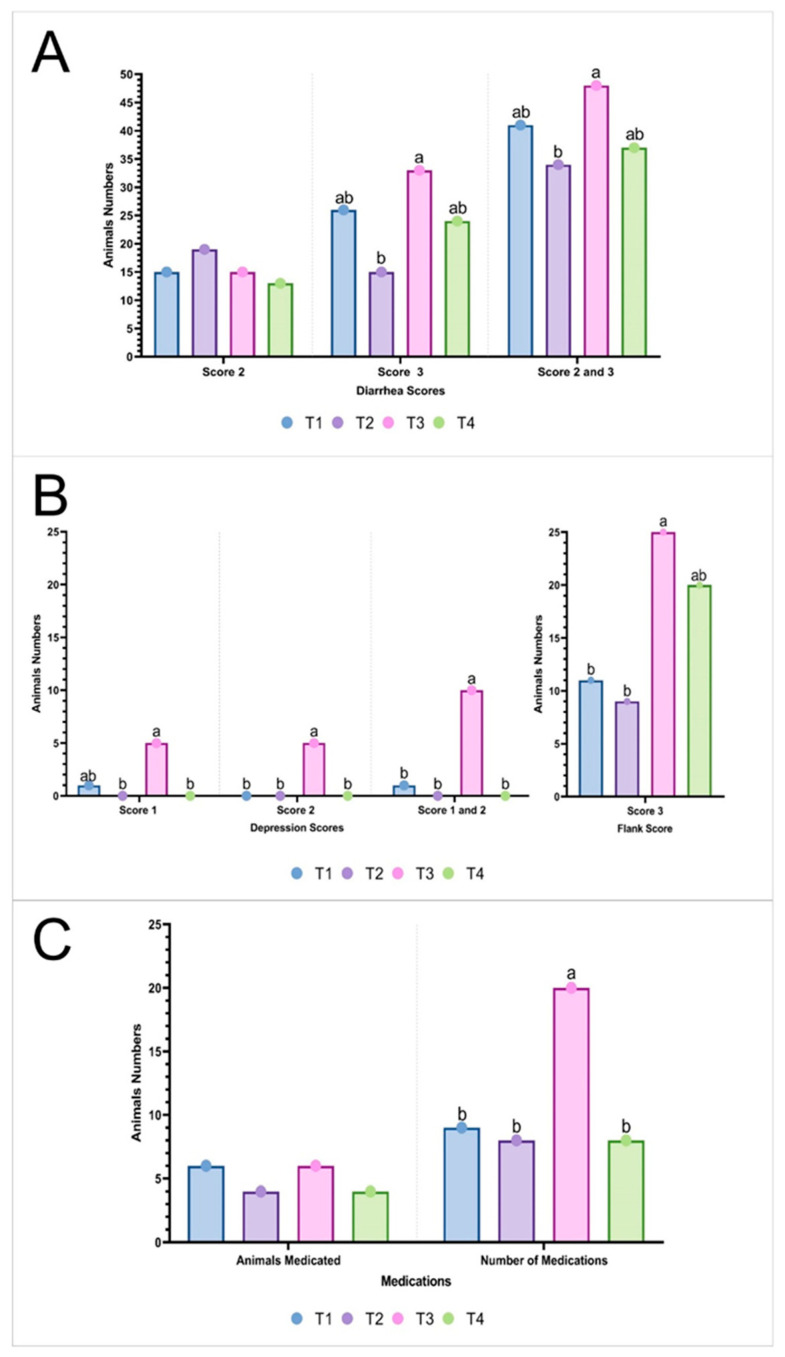
Comparative analysis of diarrheal cases (**A**), depression and flank scores (**B**), and individualized medication use (**C**) among piglets, based on different vaccination protocols (T1, T2, T3, and T4). ^a–b^ Distinct letters above the bars indicate significant differences (*p* ≤ 0.05) according to Dunn’s post hoc test, performed following analysis using the Kruskal–Wallis test. T1 received FLEXcombo^®^ (Ingelvac^®^CircoFLEX + Ingelvac^®^MycoFLEX) via intramuscular injection, followed by Enterisol^®^ Ileitis administered orally. T2 received FLEXcombo^®^ via intramuscular injection, followed by Porcilis^®^ Ileitis administered intramuscularly. T3 received Porcilis^®^ PCV M HYO via intramuscular injection, followed by Porcilis^®^ Ileitis also administered intramuscularly. T4 received FLEXcombo^®^ via intramuscular injection, followed by a 0.9% saline solution administered intramuscularly. A total of 60 piglets per treatment were evaluated.

In Figure 1A, the diarrhea scores across treatments show clear variations. For Score 3, T3 exhibited the highest number of cases, reaching 33 animals, which is more than double the number observed in T2 (15 animals), indicating a 120% increase. T1 (n = 26) and T4 (n = 24) had fewer cases, representing increases of approximately 73% and 60%, respectively, compared to T2 (*p* = 0.0098). When considering the combined Score 2 and 3 category (*p* = 0.0411), T3 again had the highest number, with 48 animals, which is a 41% increase compared to T2 (34 animals). T1 and T4 showed similar results, with 41 and 37 animals, respectively, representing increases of approximately 20% and 8% compared to T2.

The level of depression (Figure 1B) was higher in the animals in T3 compared to the other groups (*p* < 0.05). For Depression Score 1, T3 had five animals compared to just one in T1 and none in T2 and T4 (*p* = 0.0087). Similarly, for Depression Score 2, T3 had five animals, while T1, T2, and T4 had none (*p* = 0.0015). When combining Depression Scores 1 and 2, T3 showed a total of 10 animals with depression, which is 10 times higher than in T1, which had only one animal, and there were no cases in T2 and T4 (*p* < 0.0000). Regarding Flank Score 3 (Figure 1B), which indicates severely lean and empty flank piglets, T3 had a higher number of cases, with 25 animals. This represents an increase of 177% compared to T2 (nine animals) and 127% compared to T1 (11 animals; *p* = 0.0024).

Considering the variables related to animal health, the number of animals that required individualized therapeutic medications (Figure 1C) showed no differences between treatments (*p* = 0.8320). However, when analyzing the total number of medications administered, it was observed that the animals in treatment T3 received more frequent therapeutic interventions (n = 20) compared to the other groups: T1 (n = 9), T2 (n = 8), and T4 (n = 8) (*p* = 0.0104). This accounted for a notable increase, with T3 representing a 150% increase compared to T2 and T4 and a 122% increase compared to T1.

The animals vaccinated against *L. intracellularis* in T2 and T3 maintained a mean rectal temperature equal to or higher than 39.69 °C in the post-vaccination periods (Table 3). In contrast, the piglets from the T1 group showed lower temperatures (*p* > 0.05) 8 h and 24 h after vaccine application. The post-vaccine values of T1 and T4 were similar.

### 3.2. Behavior

**Figure 2:**
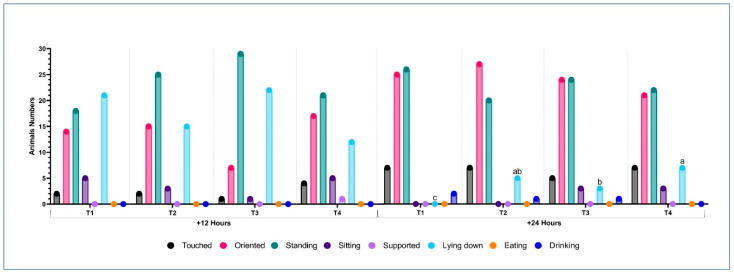
Behavioral and postural responses of piglets 12 and 24 h post-vaccination against porcine circovirus type 2 (PCV2) and *Mycoplasma hyopneumoniae* (Mhp), subjected to different vaccination protocols. ^a–c^ Distinct letters above the bars indicate significant differences (*p* ≤ 0.05) according to Dunn’s post hoc test, performed following analysis using the Kruskal–Wallis test. T1 received FLEXcombo^®^ (Ingelvac^®^CircoFLEX + Ingelvac^®^MycoFLEX) via intramuscular injection, followed by Enterisol^®^ Ileitis administered orally. T2 received FLEXcombo^®^ via intramuscular injection, followed by Porcilis^®^ Ileitis administered intramuscularly. T3 received Porcilis^®^ PCV M HYO via intramuscular injection, followed by Porcilis^®^ Ileitis also administered intramuscularly. T4 received FLEXcombo^®^ via intramuscular injection, followed by a 0.9% saline solution administered intramuscularly. A total of 60 piglets per treatment were evaluated.

Considering the behavior and posture of the piglets after vaccination against PCV2 and Mhp, it was not possible to observe differences (*p* > 0.05) among the treatments in the first 12 h after vaccination (Figure 2). At 24 h post-vaccination, no animals were observed lying down in treatment T1 (n = 0), whereas the number of lying animals was greater in the other treatments (T2: n = 5; T3: n = 3; and T4: n = 7) (*p* < 0.05).

Regarding the behavioral and postural parameters (Table 4), there was no difference (*p* ≥ 0.05) between the groups, considering the pre-vaccination periods and 72 h post-vaccination against *L. intracellularis*. Four hours after vaccine application, the number of animals touched was on average 29% lower in the groups that received treatments T2 and T3 (*p* < 0.05), indicating their refutation of the approach and acceptance of contact by humans, a behavior that signals discomfort. Additionally, in the same period, it was possible to observe that the number of animals lying down was 2.67, 4.67, and 2 times higher (*p* < 0.05) in T2, T3, and T4, respectively, compared to the animals in T1.

Evaluating the behavior and posture 12 h after the application of the *L. intracellularis* vaccine (Table 4), there was a greater number (*p* < 0.05) of animals touched in T1 and T4 (2, 4, and 4 times higher than T2 and T3, respectively). Only 13.33% of the piglets in T1 were lying down compared to 35 and 23.33% in T2 and T3, respectively (*p* < 0.05).

Twenty-four hours after vaccination, the lowest number of animals that showed sitting behavior (*p* > 0.05) was observed in T3 (5.0%); in contrast, 48 h after vaccination, approximately 41.67% of the animals with this posture belonged to the T3 group, which was higher (*p* < 0.05) compared to the other treatments.

The results of the serum levels of C-reactive protein and haptoglobin (Table 5) indicate that, in the pre-vaccination condition, as predicted, there was no difference between treatments for these parameters (*p* ≥ 0.05). However, in the two post-vaccination periods, the animals in T3 showed, for both indicators, higher values (*p* < 0.05) compared to T1 and T4. For the evaluation of haptoglobin, 48 h after vaccination, T1 showed a lower mean value than T4 (*p* < 0.05).

## 4. Discussion

International, political, and commercial agreements have been adopted in recent years, aiming to promote high standards of animal welfare worldwide [36], an attribute that is part of sustainability [37]. From the basic premises of animal welfare, pigs need to be able to live according to their behavioral needs; have the ability to express emotions; and have basic health, growth, and normal functioning of physiological systems [38]. In this context, the use of vaccines for disease prevention has become a key part of pig health management [39].

Numerous commercial vaccines are available to avoid productivity losses, inducing an effective immune response. However, vaccination can be considered as a stressful and painful event for animals caused by handling and possible adverse reactions after vaccination [28]. Vaccines for the same pathogen can cause different adverse reactions, according to the complexity of their components [30]. Thus, it is important and ethical to use effective and low-reactive vaccines in pigs to provide better animal welfare.

The scientific evaluation of vaccine reactions determined by different commercial immunizers is uncommon because the primary focus of these studies usually involves the identification of their immune efficiencies, i.e., the level and persistence of their vaccine responses, as described in the studies with the pathogens PCV2, Mhp, and *L. intracellularis* [40,41,42,43,44,45]. The evaluation of the possible discomfort of the animal is negative for the zootechnical performance that may be temporarily or persistently compromised, but it is also negative for the man who observes its suffering [20,46].

In this study, it was possible to observe some systemic adverse reactions with a negative impact on animal welfare and, consequently, on zootechnical indices, especially on daily feed intake. These findings may be associated with the fact that certain adjuvants trigger the innate response, which is necessary to optimize adaptive responses. Thus, by stimulating innate immunity, adjuvants also promote inflammation, which can be immediate after vaccination, determining transient local inflammatory reactions. Responses can also persist for a few days, with depression, fever, and worsening well-being resulting from extravasation of cytokines present at the injection site that enter the circulatory system and act in the brain [20].

However, extreme conditions linked to the reactions that could cause death were not recorded. Regarding the animals that presented clinical symptoms, it was possible to identify that a greater number of individualized medications (doses) were used in the T3 animals, with a higher prevalence of respiratory problems. According to Madapong et al. [47], immune responses following the administration of vaccines against porcine reproductive and respiratory syndrome virus (PRRSV) and PCV2, such as those used in groups T1, T2, and T4, are elevated during the first weeks post-vaccination, with antibodies and genomic copies being detected at 7, 14, and 21 days post-vaccination. This supports the observation that the immune response after the first administration of the Mhp and PCV2 vaccines is heightened in the first 14 days after vaccination. This condition may have been positive for minimizing the cases of pneumonia caused by Mhp in these experimental groups, as reported by Witvliet et al. [48]. Additionally, the behavioral differences of the animals in T3 suggest greater adverse reactions after vaccination; consequently, the pigs demonstrated less interest in eating, greater depression, and empty flanks, making them more vulnerable to illness.

According to the adjuvant (mineral oil used in the vaccine of the T2 and T3 groups), the physiological response of a vaccine can cause behavioral changes in illness, such as febrile responses, lethargy, and decreased appetite and thirst [30]. There is evidence that vaccines that induce less intense hyperthermia responses cause less energy demand by the body, resulting in milder inflammatory reactions and better adaptation and animal welfare [21]. In our study, after the application of the *L. intracellularis* vaccine, the animals in T2 and T3 showed hyperthermia eight hours after application. Despite this increase in temperature ceasing after 24 h, the piglets in T1 showed lower rectal temperatures throughout the evaluation period. It can be noted that the live attenuated vaccine, referring to the T1 treatment product, resulted in a lower systemic inflammatory reaction and, consequently, lower rectal temperatures [42,49]. These findings are typical when intramuscular vaccines are compared in which the adjuvant is based on mineral oil (T2 and T3) versus an oral attenuated vaccine based on a water adjuvant (T1).

In response to hyperthermia, animals may exhibit some behavioral patterns, such as depression, inactivity, sleepiness, and anorexia [50]. The animals in T1 had a higher percentage of active piglets, classified as touched and oriented, as well as a lower percentage of animals classified as lying at 4 and 12 h after the application of the vaccine against *L. intracellularis*. Although the number of animals that remained seated in T1 was similar to the other groups 24 h after vaccination, after this period (48 h after vaccination), the number was significantly lower (*p* > 0.05). Thus, the lower rectal temperature observed in the T1 group could indicate the better welfare of these animals, providing greater numbers of touched and oriented animals and smaller numbers of sitting animals. Additionally, in this phase of this study, the objective of the injected saline solution (T4) was to relate the animal behavior to the effect of the vaccine or the management of piglet containment. The behavioral similarity between the T1 and T4 groups may be related to the better well-being promoted by the T1 group’s vaccine due to its immunological mode of action and not to its effective administration (T1 Li vaccine drench vs. T4 saline injection).

Although the rectal temperature of the piglets subjected to vaccination against Mhp and PCV2 was not measured in this study, there are reports in the literature of hyperthermia states for animals vaccinated with the commercial vaccine used in the T3 group and normal rectal temperatures for animals vaccinated with Mhp and PCV2, the commercial vaccine used in groups T1, T2, and T4 [21,48]. These references are in line with some behavioral patterns related to the state of hyperthermia in the animals that received treatment T3, such as a higher depression index and a higher number of severely lean piglets and empty flanks. This suggests that there is a relationship between the adjuvants with these findings, although all vaccines presented the same antigens; that is, they are subunit vaccines based on the PCV2a capsid protein expressed in the baculovirus system and Mycoplasm bacterin [51].

Relative to vaccines against Mhp and PCV2, different adjuvants can cause different immune responses and different adverse effects [22]. The vaccines in groups T1, T2, and T4 contained the adjuvant ImpranFLEX^®^, an aqueous-based polymer, which has the ability to slowly trap and release antigenic molecules, stimulating immune cells [52,53]. The vaccine administered to the T3 group used the adjuvant Emunade™, an oil-in-water emulsion and aluminum hydroxide [54]. This adjuvant acts by promoting cell lysis at the application site to stimulate the immune response, resulting in a local inflammatory response by cytokines at the injection site that fall into the circulatory system and act on the brain, causing systemic changes [46]. Thus, the high level of circulating cytokines in the bloodstream due to the mode of action of the adjuvant of the T3 treatment vaccine may have caused a worsening of animal welfare. As a consequence, the rate of depressed animals increased, their willingness to eat decreased, and the number of severely lean piglets with empty flanks increased.

Thus, although vaccines against PCV2 and Mhp are historically reactive, it is important to consider immunization protocols that cause less harm to animal welfare [30,55]. In this sense, the behavior of pigs after vaccination against PCV2 and Mhp with Ingelvac^®^CircoFLEX and/or Ingelvac^®^MycoFLEX (Boehringer Ingelheim Vetmedica Inc., St. Joseph, MO, USA) has been identified with this objective, where the piglets were more oriented, allowed touching, stood more, and ate more, demonstrating a greater level of welfare. These past study results corroborate with this study, in which the pigs treated with FLEXcombo^®^, represented by the mixture of Ingelvac^®^CircoFLEX and Ingelvac^®^MycoFLEX vaccines, presented better indices of welfare.

Additionally, the disposition of piglets eating after vaccination provides an indication of animal welfare [31]. The T1 animals showed a daily feed intake 20 g higher and an average increase in final weights of 528 g when compared to the T3 group, a difference of 2.17% for the latter parameter. In this perspective, the animals from T3 showed greater anorexia, which negatively affected zootechnical performance. There are few reports comparing the performance of piglets subjected to commercial vaccines against Mhp and PCV2. However, Chae [56], in a survey that addresses the efficiency of commercial vaccines against PCV2, describes a high efficacy against the agent based on clinical, virological, immunological, and pathological evaluation under experimental and field conditions. This author notes that there are, among the immunizers available on the market, products that compromise feed consumption. This finding is completely in agreement with our results, in which the T3 group had lower consumption of feed in the first days after weaning (Table 2).

Regarding the serum levels of CRP and haptoglobin, the pre-vaccination results show that the animals were in similar health conditions and were, therefore, able to receive the vaccine treatments. The values at the two post-vaccination times (24 and 48 h) confirm the consequences of the different vaccines. The pigs in the T3 group had significantly higher levels (*p* < 0.05) than those in the other treatments evaluated (T1 and T4) (Table 5). These differences between treatments validate the use of biomarkers to monitor the different responses that vaccines/adjuvants can initiate on acute phase proteins [25]. Variations in the inflammatory responses, according to Hernández-Caravaca et al. [21] who evaluated commercial vaccines for Mhp prevention, are related to the volume administered and the adjuvant characteristic. These authors observed that the highest levels of CRP and haptoglobin of the most reactive vaccines evaluated were associated with increased body temperature of the animals, confirming our findings. Stress conditions after weaning are favorable to increased serum concentrations of inflammation biomarkers. Therefore, vaccines that have a lower inflammatory response should be considered to not potentiate these effects [21], as higher levels of CRP and haptoglobin are associated with poorer performance in pigs [57]. A negative aspect that was also present in our study was the reduction in the consumption of ration in the pre-initial phase II, precisely the period that followed this vaccination. This proved the adverse effect of the vaccination with the T3 product when compared with T1 and T4 (Table 5).

The animals in the group that received the T1 treatment showed better well-being and, consequently, greater willingness to approach the feed and eat in addition to lower depression and empty flanks, aspects that reflected better zootechnical performance. However, the vaccination protocol of treatment T3 caused greater adverse reactions, with a negative impact on animal welfare and worsening of the zootechnical indices. These are important considerations when evaluating vaccine options and the important variable of animal welfare. It is important to note, however, that this study did not measure immune responses against the pathogens or include challenge tests, and therefore, the results do not indicate which commercial vaccine is superior in terms of pathogen control.

## 5. Conclusions

Based on this study’s results, vaccination protocols had varying impacts on piglet welfare and performance during the nursery phase. The T3 group, which received the Porcilis^®^ PCV M HYO vaccine, exhibited more adverse reactions, including higher diarrhea scores, increased incidence of depression, and a higher number of medications administered. This group also showed the highest serum levels of C-reactive protein and haptoglobin, indicating a stronger inflammatory response.

In contrast, the T1 group, which received FLEXcombo^®^ and Enterisol^®^ Ileitis, demonstrated the best welfare outcomes, with fewer animals exhibiting signs of illness or requiring medical intervention. The piglets in T1 also had higher feed intake and better overall performance compared to those in the T3 group. These findings suggest that, while all vaccination protocols resulted in comparable health and performance outcomes, the T1 protocol offered the most favorable balance between welfare indicators, inflammatory response, and overall animal comfort, making it the most suitable option for improving piglet welfare during the nursery phase.

## Figures and Tables

**Table 1:** Experimental design with vaccination strategies for Porcine Circovirus Type 2 (PCV2), *Mycoplasma hyopneumoniae* (Mhp), and *Lawsonia intracellularis* across different treatment groups, including antigen details and adjuvants.

Treatments	T1	T2	T3	T4
**Antigen**	**Vaccination I (22 days old)**			
PCV2 and Mhp *	FLEXcombo^®^		Porcilis^®^ PCV M HYO	FLEXcombo^®^
**Dose**	Single dose (2 mL)	Single dose (2 mL)	Single dose (2 mL)	Single dose (2 mL)
**Route**	IM	IM	IM	IM
**Antigen**	Vaccination II (29 days old)			
***L. intracellularis***	Enterisol^®^ Ileitis	Porcilis^®^ Ileitis	Porcilis^®^ Ileitis	Saline solution 0.9%
**Dose**	Single dose (2 mL)	Single dose (2 mL)	Single dose (2 mL)	Single dose (2 mL)
**Route**	Oral (by drench)	IM	IM	IM
**Vaccine details**				
**Vaccine**	Antigen		Adjuvant	
FLEXcombo^®^	Porcine circovirus type 2 ORF2 protein and		^‡^ Carbomer (polymer compound of acrylic acid)	
	*Mycoplasma hyopneumoniae* bacterin			
Enterisol Ileitis^®^	Live attenuated *Lawsonia intracellularis*		No adjuvant	
Porcilis PCV M HYO^®^	Porcine circovirus type 2 (PCV2) ORF2 subunit antigen and *Mycoplasma hyopneumoniae* bacterin		^ϕ^ Light mineral oil and aluminum (as hydroxide)	
Porcilis ileitis^®^	*Lawsonia intracellularis* bacterin		Light mineral oil and vitamin E-acetate	

* FLEXcombo^®^ = Ingelvac^®^CircoFLEX + Ingelvac^®^MycoFLEX; and Enterisol^®^ Ileitis (Boehringer Ingelheim Vetmedica Inc., St. Joseph, MO, USA). The vaccines for PCV2 and Mhp are mixed at the time of application, as recommended by the manufacturer. Porcilis^®^ PCV M HYO and Porcilis^®^ Ileitis (MSD Animal Health, Boxmeer, the Netherlands). IM = intramuscular. ^‡^ ImpranFLEX^®^ (Boehringer Ingelheim Vetmedica Inc., St. Joseph, MO, USA); ^ϕ^ Emunade™ (MSD Animal Health, Boxmeer, the Netherlands).

**Table 2:** Growth performance and feed efficiency of piglets during nursery phase under different vaccination protocols.

Parameters	Treatments				CV (%)	*p*-Value
	T1 (* n = 10)	T2 (n = 10)	T3 (n = 10)	T4 (n = 10)		
	Pre-initial I (21–28 d)					
BW21d (kg)	6.344 ± 0.29	6.371 ± 0.28	6.342 ± 0.28	6.345 ± 0.28	13.55	0.9998
DWG (kg)	0.130 ± 0.01	0.125 ± 0.01	0.107 ± 0.01	0.113 ± 0.01	31.36	0.5209
DFI (kg)	0.195 ± 0.01	0.200 ± 0.01	0.182 ± 0.01	0.186 ± 0.01	15.40	0.5436
FCR	1.604 ± 0.11	1.711 ± 0.17	1.730 ± 0.14	1.703 ± 0.12	25.07	0. 6979
	Pre-initial II (29–35 d)					
BW29d (kg)	7.254 ± 0.30	7.253 ± 0.26	7.092 ± 0.26	7.143 ± 0.27	11.60	0.9673
DWG (kg)	0.290 ± 0.01	0.264 ± 0.01	0.244 ± 0.01	0.275 ± 0.01	15.75	0.1108
DFI (kg)	0.456 ^a^ ± 0.01	0.437 ^ab^ ± 0.01	0.397 ^b^ ± 0.01	0.450 ^ab^ ± 0.01	11.16	0.0258
FCR	1.579 ± 0.03	1.672 ± 0.05	1.641 ± 0.05	1.667 ± 0.08	11.44	0.6913
	Initial I (36–42 d)					
BW36d (kg)	9.285 ± 0.36	9.106 ± 0.35	8.806 ± 0.28	9.081 ± 0.30	10.99	0.7791
DWG (kg)	0.441 ± 0.02	0.452 ± 0.01	0.429 ± 0.01	0.438 ± 0.01	10.88	0.7610
DFI (kg)	0.773 ± 0.02	0.769 ± 0.02	0.747 ± 0.01	0.772 ± 0.01	8.32	0.7870
FCR	1.764 ± 0.03	1.701 ± 0.02	1.745 ± 0.02	1.768 ± 0.03	5.24	0.3568
	Initial II (43–63 d)					
BW42d (kg)	15.467 ± 0.58	15.447 ± 0.54	14.815 ± 0.35	15.215 ± 0.43	9.76	0.7641
DWG (kg)	0.664 ± 0.01	0.650 ± 0.01	0.673 ± 0.01	0.697 ± 0.02	8.33	0.3125
DFI (kg)	1.041 ± 0.02	1.047 ± 0.02	1.042 ± 0.03	1.064 ± 0.05	10.36	0.9661
FCR	1.572 ± 0.03	1.625 ± 0.07	1.546 ± 0.03	1.528 ± 0.05	10.94	0.6362
BW63d (kg)	24.769 ± 0.62	24.551 ± 0.72	24.241 ± 0.33	24.976 ± 0.68	7.54	0.8513
	Total (22–64 d)					
DWG (kg)	0.438 ± 0.01	0.432 ± 0.01	0.426 ± 0.01	0.443 ± 0.01	7.39	0.6759
DFI (kg)	0.713 ± 0.01	0.711 ± 0.01	0.693 ± 0.01	0.718 ±0.02	6.60	0.6674
FCR	1.630 ± 0.02	1.654 ± 0.04	1.627 ± 0.02	1.618 ± 0.02	5.36	0.8403

^a–b^ Distinct letters within the rows indicate significant differences (*p* < 0.05) according to Tukey’s post hoc test, performed following analysis using the General Linear Model. T1 received FLEXcombo^®^ (Ingelvac^®^CircoFLEX + Ingelvac^®^MycoFLEX) via intramuscular injection, followed by Enterisol^®^ Ileitis administered orally. T2 received FLEXcombo^®^ via intramuscular injection, followed by Porcilis^®^ Ileitis administered intramuscularly. T3 received Porcilis^®^ PCV M HYO via intramuscular injection, followed by Porcilis^®^ Ileitis also administered intramuscularly. T4 received FLEXcombo^®^ via intramuscular injection, followed by a 0.9% saline solution administered intramuscularly. CV: coefficient of variation; BW: body weight; DWG: daily weight gain; DFI: daily feed intake; FCR: feed conversion ratio; * pen with 6 piglets each as experimental unit; ± standard error of mean.

**Table 3:** Rectal temperature of piglets before and after vaccination against *L. intracellularis* under different treatment protocols.

Temperature (°C)	Treatments				CV (%)	*p*-Value
	T1 (* n = 20)	T2 (n = 20)	T3 (n = 20)	T4 (n = 20)		
−1 h	39.11 ± 0.24	39.17 ± 0.25	39.19 ± 0.22	39.15 ± 0.24	1.03	0.9296
+8 h	39.59 ^b^ ± 0.28	40.77 ^a^ ± 0.57	40.90 ^a^ ± 0.48	39.51 ^b^ ± 0.27	1.18	0.0000
+24 h	39.44 ^c^ ± 0.21	39.81 ^a^ ± 0.30	39.69 ^ab^ ± 0.34	39.47 ^bc^ ± 0.29	0.89	0.0031

^a–c^ Distinct letters within the rows indicate significant differences (*p* ≤ 0.05) and trends (0.05 < *p* ≤ 0.10) according to Tukey’s post hoc test, performed following analysis using the General Linear Model (GLM). T1 received FLEXcombo^®^ (Ingelvac^®^CircoFLEX + Ingelvac^®^MycoFLEX) via intramuscular injection, followed by Enterisol^®^ Ileitis administered orally. T2 received FLEXcombo^®^ via intramuscular injection, followed by Porcilis^®^ Ileitis administered intramuscularly. T3 received Porcilis^®^ PCV M HYO via intramuscular injection, followed by Porcilis^®^ Ileitis also administered intramuscularly. T4 received FLEXcombo^®^ via intramuscular injection, followed by a 0.9% saline solution administered intramuscularly. CV: coefficient of variation. ± standard deviation. * A total of 20 piglets per treatment were evaluated.

**Table 4:** Behavioral responses and postures of piglets at various timepoints before and after vaccination with different immunization protocols against *L. intracellularis*.

			Oriented		Not Oriented					
	Treatments *		Touched	Oriented	Standing	Sitting	Supported	Lying Down	Eating	Drinking
Before Vaccination	**T1**	**n**	3	7	24	7	0	19	0	0
		**%**	5.00	11.67	40.00	11.67	0.00	31.67	0.00	0.00
	**T2**	**n**	6	10	19	4	0	19	0	2
		**%**	10.00	16.67	31.67	6.67	0.00	31.67	0.00	3.33
	**T3**	**n**	5	14	21	10	0	10	0	0
		**%**	8.33	23.33	35.00	16.67	0.00	16.67	0.00	0.00
	**T4**	**n**	2	13	16	10	0	19	0	0
		**%**	3.33	21.67	26.67	16.67	0.00	31.67	0.00	0.00
	***p*-value**		0.48	0.44	0.64	0.36	--	0.3	--	0.11
+4 h	**T1**	**n**	8 ^b^	22	21	6	0	3 ^c^	0	0
		**%**	13.33	36.67	35.00	10.00	0.00	5.00	0.00	0.00
	**T2**	**n**	3 ^c^	12	25	12	0	8 ^b^	0	0
		**%**	5.00	20.00	41.67	20.00	0.00	13.33	0.00	0.00
	**T3**	**n**	2 ^c^	18	19	7	0	14 ^a^	0	0
		**%**	3.33	30.00	31.67	11.67	0.00	23.33	0.00	0.00
	**T4**	**n**	11 ^a^	18	21	4	0	6 ^b^	0	0
		**%**	18.33	30.00	35.00	6.67	0.00	10.00	0.00	0.00
	***p*-value**		0.02	0.41	0.83	0.19	--	0.04	--	0.00
+12 h	**T1**	**n**	1	12 ^a^	32	7	0	8 ^c^	0	0
		**%**	1.67	20.00	53.33	11.67	0	13.33	0.00	0.00
	**T2**	**n**	0	5 ^b^	26	8	0	21 ^a^	0	0
		**%**	0	8.33	43.33	13.33	0	35.00	0.00	0.00
	**T3**	**n**	0	3 ^b^	34	9	0	14 ^b^	0	0
		**%**	0	5.00	56.67	15	0	23.33	0.00	0.00
	**T4**	**n**	2	12 ^a^	39	6	1	0 ^d^	0	0
		**%**	3.33	20.00	65.00	10.00	1.67	0.00	0.00	0.00
	***p*-value**		0.3	0.04	0.45	0.88	0.39	0	--	--
+24 h	**T1**	**n**	4	14	14	8 ^ab^	0	13	0	0
		**%**	6.67	23.33	35.00	13.33	0.00	21.67	0.00	0.00
	**T2**	**n**	2	10	24	12 ^a^	0	10	0	2
		**%**	3.33	16.67	16.67	20.0	0.00	16.67	0.00	3.33
	**T3**	**n**	3	11	26	3 ^b^	0	17	0	0
		**%**	5.00	18.33	18.33	5.00	0.00	28.33	0.00	0.00
	**T4**	**n**	3	15	18	13 ^a^	0	11	0	0
		**%**	5.00	25.00	25.00	21.67	0.00	18.33	0.00	0.00
	***p*-value**		0.88	0.71	0.65	0.08	--	0.52	--	0.21
+48 h	**T1**	n	9	13	20	5 ^c^	0	13	0	0
		**%**	15.00	21.67	33.33	8.33	0.00	21.67	0.00	0.00
	**T2**	**n**	3	11	24	6 ^c^	0	16	0	0
		**%**	5.00	18.33	40.00	10.00	0.00	26.67	0.00	0.00
	**T3**	**n**	7	12	12	25 ^a^	0	15	0	0
		**%**	11.67	20	20.00	41.67	0.00	25	0.00	0.00
	**T4**	**n**	7	15	12	13 ^b^	0	11	0	1
		**%**	11.86	25.42	20.34	22.03	0.00	18.64	0.00	1.69
	***p*-value**		0.4	0.86	0.18	0.01	--	0.8	--	0.51
+72 h	**T1**	**n**	11	19	25	0	0	5	0	0
		**%**	18.33	31.67	41.67	0.00	0.00	8.33	0.00	0.00
	**T2**	**n**	14	14	26	3	0	2	0	1
		**%**	23.33	23.33	43.33	5.00	0.00	3.33	0.00	1.67
	**T3**	**n**	11	17	24	3	0	1	0	1
		**%**	18.64	28.81	40.68	5.08	0.00	1.69	0.00	1.69
	**T4**	**n**	17	21	12	4	0	3	0	2
		**%**	28.81	35.59	20.34	6.78	0.00	5.08	0.00	3.39
	***p*-value**		0.59	0.66	0.13	0.77	--	0.7	--	0.8

^a–d^ Distinct letters within the rows indicate significant differences (*p* ≤ 0.05) according to Dunn’s post hoc test, performed following analysis using the Kruskal–Wallis test. T1 received FLEXcombo^®^ (Ingelvac^®^CircoFLEX + Ingelvac^®^MycoFLEX) via intramuscular injection, followed by Enterisol^®^ Ileitis administered orally. T2 received FLEXcombo^®^ via intramuscular injection, followed by Porcilis^®^ Ileitis administered intramuscularly. T3 received Porcilis^®^ PCV M HYO via intramuscular injection, followed by Porcilis^®^ Ileitis also administered intramuscularly. T4 received FLEXcombo^®^ via intramuscular injection, followed by a 0.9% saline solution administered intramuscularly. * A total of 60 piglets per treatment were evaluated.

**Table 5:** Inflammatory markers (C-reactive protein and haptoglobin) in piglets subjected to different vaccination protocols against *L. intracellularis*.

Parameters	Treatments			CV (%)	*p*-Value
	T1 (* n = 20)	T3 (* n = 20)	T4 (* n = 20)		
1 h before vaccination					
CRP (ng/mL)	479.5 ± 75.3	417.7 ± 55.3	529.3 ± 72.2	63.26	0.5253
Haptoglobin (ng/mL)	59.1 ± 25.2	75.6 ± 16.3	37.2 ± 9.3	99.8	0.1780
24 h post-vaccination					
CRP (ng/mL)	549.5 ^b^ ± 79.3	1034.8 ^a^ ± 112.4	526.6 ^b^ ± 526.6	63.19	0.0001
Haptoglobin (ng/mL)	58.4 ^b^ ± 17.3	535.1 ^a^ ± 95.8	176.8 ^b^ ± 58.5	124.3	0.0004
48 h post-vaccination					
CRP (ng/mL)	643.9 ^ab^ ± 86.3	894.3 ^a^ ± 83.5	421.3 ^b^ ± 69.8	61.17	0.0003
Haptoglobin (ng/mL)	197.1 ^c^ ± 56.4	979.3 ^a^ ± 89.2	494.1 ^b^ ± 75.7	79.3	0.0000

^a–c^ Distinct letters within the rows indicate significant differences (*p* ≤ 0.05) and trends (0.05 < *p* ≤ 0.10) according to Tukey’s post hoc test, performed following analysis using the General Linear Model (GLM). T1 received FLEXcombo^®^ (Ingelvac^®^CircoFLEX + Ingelvac^®^MycoFLEX) via intramuscular injection, followed by Enterisol^®^ Ileitis administered orally. T2 received FLEXcombo^®^ via intramuscular injection, followed by Porcilis^®^ Ileitis administered intramuscularly. T3 received Porcilis^®^ PCV M HYO via intramuscular injection, followed by Porcilis^®^ Ileitis also administered intramuscularly. T4 received FLEXcombo^®^ via intramuscular injection, followed by a 0.9% saline solution administered intramuscularly. CV: coefficient of variation; ± standard error of mean; * A total of 20 piglets per treatment were evaluated; CRP: C-reactive protein.

## Data Availability

The datasets generated during and/or analyzed during the current study are available from the corresponding author on reasonable request.

## Supplementary Materials

The following supporting information can be downloaded at: https://www.mdpi.com/article/10.3390/vaccines12111242/s1, Table S1. Parameters measured, their respective methods, and indications for piglet performance and welfare assessment.
