# Supplementary material for: Well-Being and Performance of Nursery Pigs Subjected to Different Commercial Vaccines Against Porcine Circovirus Type 2, Mycoplasma hyopneumoniae and Lawsonia intracellularis
Source: Vaccines (Basel). 2024 Oct 31;12(11):1242. doi: 10.3390/vaccines12111242 (PMC11598480; doi:10.3390/vaccines12111242)
Supplement: Supplementary file 1 [file vaccines-12-01242-s001.zip › vaccines-3237609-supplementary.pdf]

Table S1. Parameters measured, their respective methods, and indications for piglet performance and welfare assessment.

Parameter	Measurement Method	Indication
Daily Feed Intake (DFI)	Weighing of feed, subtracting leftovers and losses	Reflects the piglets' consumption of feed and appetite.
Daily Weight Gain (DWG)	Weekly individual weighing of piglets	Indicates growth and development performance.
Feed Conversion (FC)	Ratio of feed intake to weight gain	Efficiency of feed utilization for growth.
Diarrhea Score	Visual assessment of feces (0: normal, 1: pasty, 2: moderately fluid, 3: watery)	Indicates digestive health and presence of enteric issues such as diarrhea.
Depression Score	Adapted from Rossi et al. (0: lively, 1: slight isolation, 2: delayed response, 3: depressed and reluctant to rise)	Reflects the piglets' mental state, behavior, and response to environmental stimuli.
Flank Score	Visual assessment of body condition (1: full abdomen, 2: flat flanks, 3: empty flanks)	Indicates body condition, particularly signs of malnutrition or insufficient intake.
Rectal Temperature	Measurement at 1 hour before, 8 and 24 hours post-vaccination (*L. intracellularis*)	Indicates fever or inflammatory response to vaccination.
Behavior and Posture	Classified as touched, oriented, not oriented; postures: standing, sitting, supported, lying; behaviors: eating, drinking	Reflects the piglets' welfare, activity, and potential discomfort following vaccination.
Serum Haptoglobin	ELISA method, measured at pre-vaccination (D7), 24 hours (D8), and 48 hours (D9) post-vaccination	Acute phase protein, indicates systemic inflammation or immune response to stress or infection.
Serum C-Reactive Protein	ELISA method, measured at pre-vaccination (D7), 24 hours (D8), and 48 hours (D9) post-vaccination	Another acute phase protein, indicating inflammation and immune response.
